# IS*26*-mediated plasmid reshuffling results in convergence of toxin–antitoxin systems but loss of resistance genes in XDR *Klebsiella pneumoniae* from a chronic infection

**DOI:** 10.1099/mgen.0.000892

**Published:** 2022-09-28

**Authors:** Ting L. Luo, Brendan W. Corey, Erik Snesrud, Alina Iovleva, Christi L. McElheny, Lan N. Preston, Yoon I. Kwak, Jason W. Bennett, Yohei Doi, Patrick T. McGann, Francois Lebreton

**Affiliations:** ^1^​ Multidrug-Resistant Organism Repository and Surveillance Network (MRSN), Walter Reed Army Institute of Research, Silver Spring, Maryland, USA; ^2^​ Division of Infectious Diseases, University of Pittsburgh School of Medicine, Pittsburgh, Pennsylvania, USA

**Keywords:** CRE, mosaic plasmids, NDM, OXA-232, ST14, within-host evolution

## Abstract

Carbapenem-resistant *Enterobacterales* pose an urgent threat to human health worldwide. *Klebsiella pneumoniae* sequence type (ST) 14, initially identified in the Middle East and South-Asia and co-harbouring the carbapenemase genes *bla*_OXA-232_ and *bla*_NDM-1,_ is now emerging globally. One such strain was detected in the USA in 2013 from a patient initially treated in India that also carried *armA*, a 16S rRNA methyltransferase that confers resistance to all clinically relevant aminoglycosides. Genetic and phenotypic changes were observed in 14 serial isolates collected from this chronically infected patient. The index isolate carried five plasmids, including an IncFIB–IncHI1B (harbouring *armA* and *bla*_NDM-1_), an IncFIA (*bla*_CTX-M-15_) and a ColE-like (*bla*_OXA-232_), and was extensively resistant to antibiotics. Four years later, a subsequent isolate had accumulated 34 variants, including a loss-of-function mutation in *romA*, resulting in tigecycline non-susceptibility. Importantly, this isolate now only carried two plasmids, including a large mosaic molecule made of fragments, all harbouring distinct toxin–antitoxin systems, from three of the canonical plasmids. Of the original acquired antibiotic resistance genes, this isolate only retained *bla*_CTX-M-15_, and as a result susceptibility to the carbapenems and amikacin was restored. Long-read sequencing of a subset of five representative isolates, collected between 2013 and 2017, allowed for the elucidation of the complex plasmid patterns and revealed the role of IS*26*-mediated plasmid reshuffling in the evolution of this clone. Such investigations of the mechanisms underlying plasmid stability, together with global and local surveillance programmes, are key to a better understanding of plasmid host range and dissemination.

## Data Summary

Data pertinent to this study have been deposited in National Center for Biotechnology Information (NCBI) BioProject PRJNA830444. The 14 serial isolates featured in this study have been assigned BioSample accessions of SAMN27722563–SAMN27722576. Patient clinical metadata can be accessed through BioSample or Table 1 in this paper. The 14 sets of Illumina reads and 5 sets of PacBio reads have been uploaded and assigned SRA accessions SRR18887175–18887193.

**Fig. 1.**
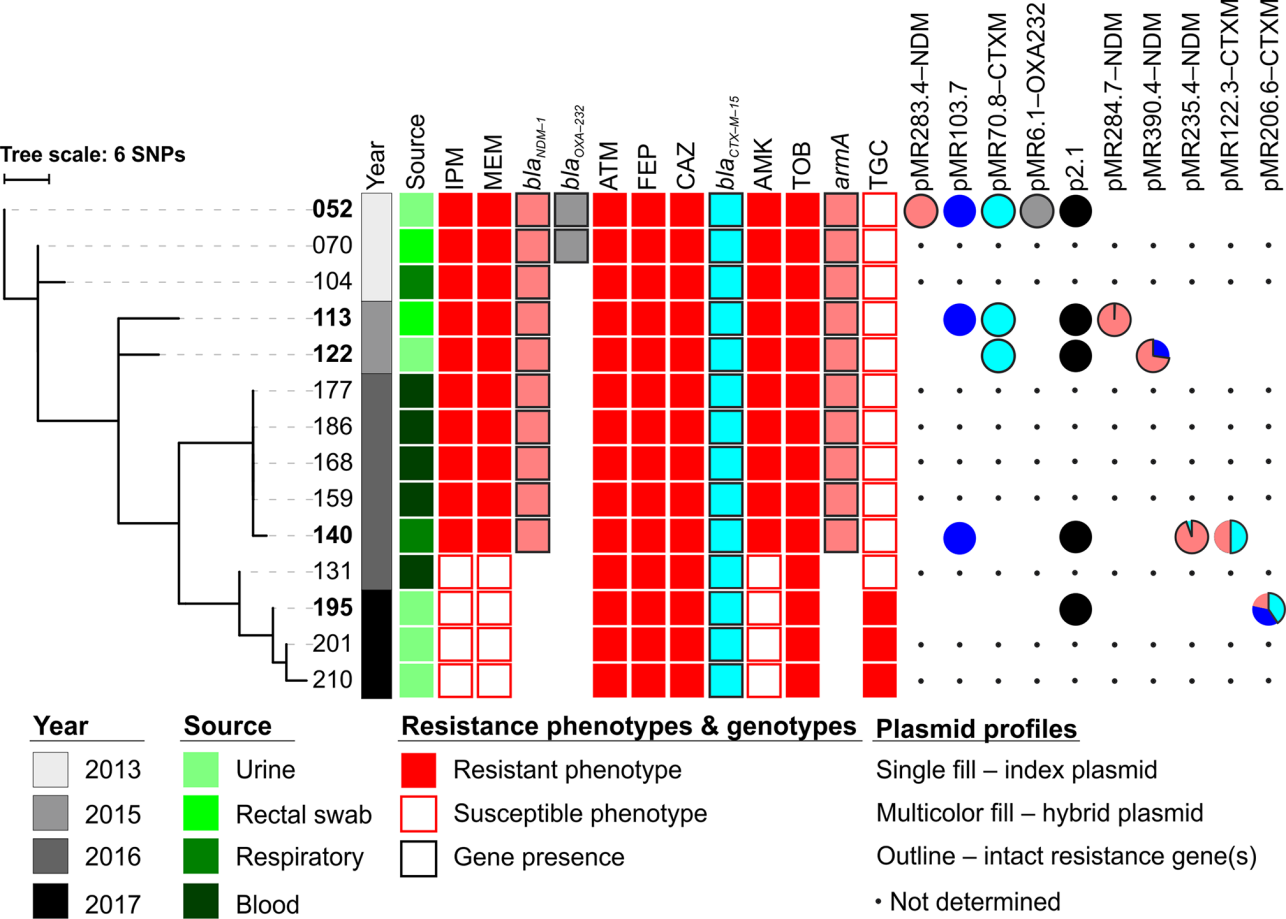
SNP-based phylogeny of 14 serial ST14 *K. pneumoniae *. For clarity, the isolate names provided in Table 1 are abbreviated to the last three digits. Phenotypic susceptibilities to select antibiotics are shown (squares) as well as the presence/absence of relevant AMR genes (box coloured so as to designate the corresponding carrying plasmid). For the five isolates with complete genomes, the presence of plasmids (named according to their size in kb and pertinent AMR genes carried) is indicated (coloured circles). Hybrid plasmids are depicted as pie charts with each segment from a canonical plasmid featured as a coloured slice (proportional to its size).

**Table 1.** Isolates and patient metadata

Isolate*	Date	Source	Documented treatment
MRSN 546**052**	6 March 2013	Urine	Fosfomycin
MRSN 546**070**	2013	Rectal swab	None
MRSN 546**104**	27 June 2013	Respiratory	None
MRSN 546**113**	15 April 2015	Rectal swab	None
MRSN 546**122**	8 August 2015	Urine	None
MRSN 546**131**	8 February 2016	Blood	Ceftazidime–avibactam, fosfomycin
MRSN 546**177**	14 June 2016	Blood	Ceftazidime–avibactam
MRSN 546**186**	14 June 2016	Blood	Ceftazidime–avibactam
MRSN 546**159**	15 June 2016	Blood	Ceftazidime–avibactam
MRSN 546**168**	15 June 2016	Blood	Ceftazidime–avibactam
MRSN 546**140**	16 June 2016	Respiratory	Ceftazidime-avibactam
MRSN 546**195**	12 June 2017	Urine	Fosfomycin
MRSN 546**201**	15 September 2017	Urine	None
MRSN 546**210**	12 November 2017	Urine	Fosfomycin

*MRSN identification number. Only the last three digits (bold font) are shown in Figs. 1 and 4.

**Fig. 2.**
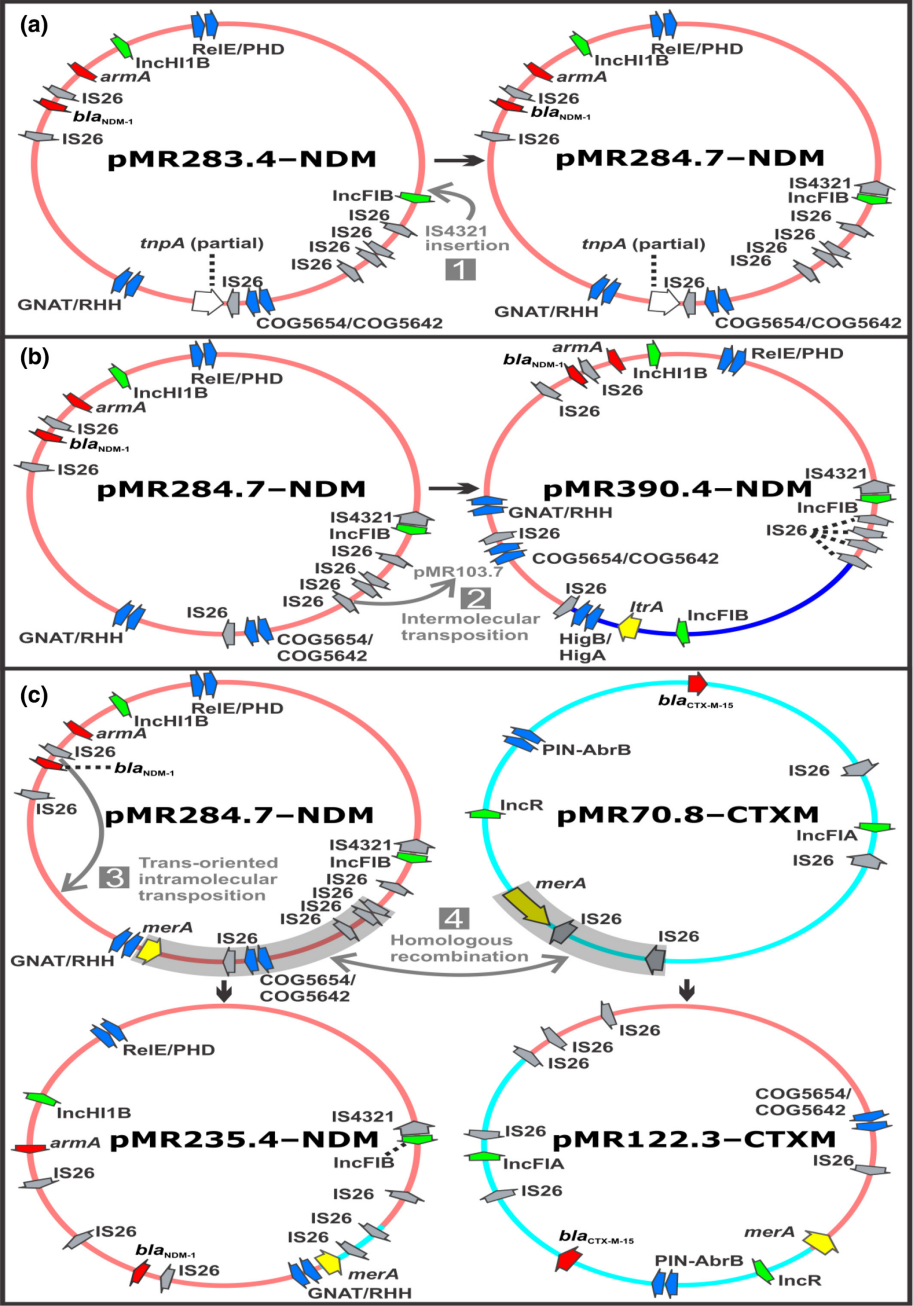
Proposed pathway for genesis of hybrid plasmids pMR284.7-NDM, pMR390.4-NDM, pMR235.4-NDM and pMR122.3-CTXM. (**a**) Index plasmid pMR283.4-NDM undergoes IS*4321* insertion (step 1) to create hybrid plasmid pMR284.7-NDM. (**b**) Hybrid plasmid pMR284.7-NDM undergoes intermolecular transposition with index plasmid pMR103.7 (step 2) to create hybrid plasmid pMR390.4-NDM. (**c**) A combination of trans-oriented intramolecular transposition (step 3) and homologous recombination (step 4) events involving hybrid plasmid pMR284.7-NDM and index plasmid pMR70.8-CTXM results in the hybrid plasmids pMR235.4-NDM and pMR122.3-CTXM. Segments of canonical plasmids are rendered according to their assigned colour in Fig. 1. Antimicrobial resistance genes (red), insertion sequences (grey), plasmid replicon ori sites (green) and putative transposases (white) are indicated, as well as genes at the boundary of recombination or insertion events (yellow). Segments involved in insertion, inversion and/or recombination are shaded (grey arrow), and directionality of event is shown (black arrow).

**Fig. 3.**
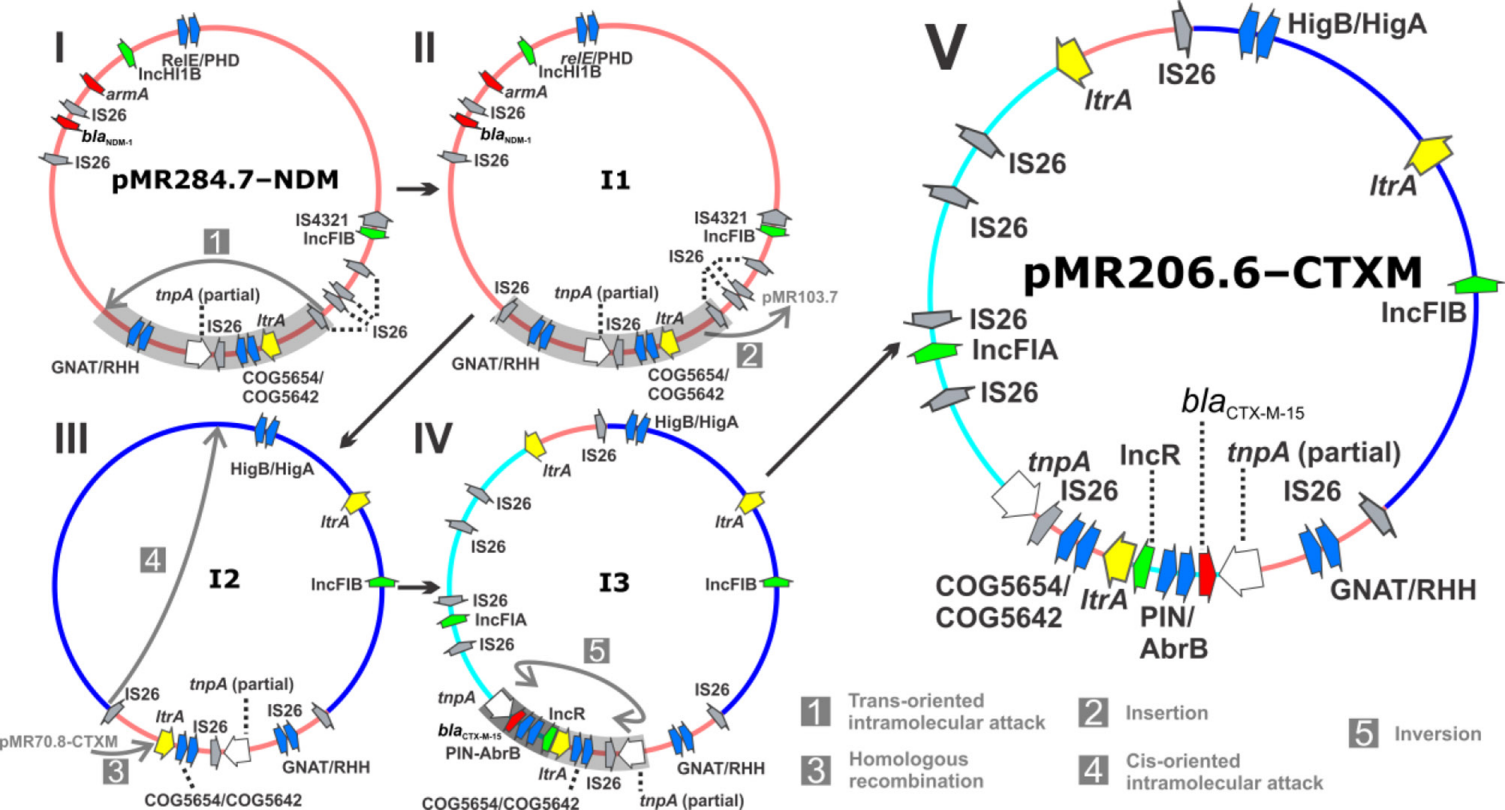
Proposed pathway for genesis of hybrid plasmid pMR206.6-CTXM. Plasmid pMR284.7-NDM experiences several events (steps 1–5) resulting in the loss of *armA* and *bla*_NDM-1_. Target site duplication motifs implicate IS*26* in the proposed pathway. Proposed (i.e. not observed) intermediary plasmid structures (I1–3) are indicated between each step. The final plasmid product is a mosaic of the three large AMR-carrying canonical plasmids in the index isolate. Segments of canonical plasmids are rendered according to their assigned colour in Fig. 1. Antimicrobial resistance genes (red), insertion sequences (grey), plasmid replicon ori sites (green) and putative transposases (white) are indicated, as well as genes at the boundary of recombination or insertion events (yellow). Segments involved in insertion, inversion and/or recombination are shaded (grey arrow), and directionality of event is shown (black arrow).

**Impact Statement**

Carbapenem-resistant *Klebsiella pneumoniae* is a significant nosocomial threat. This study provides a rare glimpse into the plasmid evolution of an extensively drug-resistant clone of *K. pneumoniae* that has persisted within a single host for an extended period of time. The source isolate harboured two carbapenemases on separate plasmids. Five years later, the same lineage of *K. pneumoniae* had lost both carbapenemases and reverted to a susceptible phenotype. Analysis of closed plasmid sequences revealed the critical role of the insertion sequence IS*26* in plasmid reshuffling. Importantly, the presence of toxin–antitoxin (TA) systems did not prevent the loss of plasmid backbones and regions carrying the resistance genes. Instead, IS*26*-mediated rearrangements allowed for the convergence of selected sequences, all carrying the TA system from their ancestral plasmids, into a single, large, mosaic molecule. Improving our knowledge of the mechanisms of plasmid plasticity and stability is key to better understand the role of these molecules, and genetic determinants within them, in the persistence and spread of associated antibiotic resistance genes.

## Introduction

Bacterial antimicrobial resistance has emerged as one of the leading public health challenges of the 21st century [1]. The global increase in carbapenem-resistant *Enterobacterales* (CRE), best illustrated by the rapid spread of ‘high-risk’ *Klebsiella pneumoniae* lineages, is universally regarded as a critical priority by international and national health agencies [2].

At the origin of the emergence of carbapenem-resistant *K. pneumoniae* (CRKP) lineages is the acquisition, mostly via conjugative plasmids, of carbapenemases, generally from one of three classes: the class A carbapenemases exemplified by the *K. pneumoniae* carbapenemases (KPCs), the class B metallo-β-lactamases (IMP, NDM and VIM) and the class D OXA-48-type oxacillinases [3]. While sequence type (ST) 258 complex (often carrying an IncFII plasmid harbouring a KPC carbapenemase) has become one of the most successful lineages of CRKP globally [4], those carrying NDM and OXA-48-type enzymes dominate in some regions of the world, including the Middle East and South-Asia [5–7]. One consequence of the high prevalence of these two groups of carbapenemases was the emergence of strains carrying both NDM-1 and OXA-48 enzymes (including its variants OXA-181 and OXA-232) [8, 9]. While problematic alone, these warrant specific surveillance as a major reservoir of non-KPC carbapenemases in an era where KPC-selective inhibitors are being used as first-line treatment options for CRKP [10].

Lineage *K. pneumoniae* ST14, which recently became prevalent in some regions of the USA [11], often carries *bla*_OXA-232_ and sometimes co-harbours *bla*_NDM-1_ [12]. In 2014, the first report of such an extensively drug-resistant (XDR) clone in the USA documented the carriage of *bla*_OXA-232_ by a ColE-like plasmid with both *bla*_NDM-1_ and the 16S rRNA methyltransferase gene *armA* on a hybrid IncFIB–IncHI1B plasmid [13, 14]. The latter shared similarities with plasmid pNDM-MAR, the representative of a plasmid family first described in a *K. pneumoniae* from Morocco in 2012 [15]. Since then, members of this family have been detected globally and have played a major role in the spread of *bla*_NDM-1_ [16, 17], including as occasional co-carriers of biomarker genes previously linked to hypervirulence [18, 19].

Contrasting with the rapid spread and increased prevalence of these carbapenemase-carrying plasmids globally, various *in vitro* and *in vivo* studies [20–22] suggest that their presence has a negative impact on the fitness of *K. pneumoniae *, possibly explaining their observed instability in the absence of selective pressure [23]. Investigations of the mechanisms underlying plasmid stability, preferentially carried out on relevant clinical specimens, are important to directly improve our understanding of the impediments and opportunities for rapid dissemination. Here, starting from the same ST14 *K. pneumoniae* strain previously carrying NDM-1 and OXA-232 [13, 14], we trace the genetic and phenotypic (AST) changes observed in 14 serial ST14 isolates collected from the same patient over the span of 5 years.

## Methods

### Bacterial isolates

The index isolate PittNDM01 (hereafter named MRSN 546052 to reflect the exact isolate sequenced in this study) was previously characterized as sequence type 14 (ST-14) and coproducing NDM-1 and OXA-232 [13, 14]. For this study, the complete set of 14 serial *K. pneumoniae* isolates from the same patient were collected throughout multiple visits at the same hospital system in Pittsburgh (PA) (Table 1). Bacterial species identification and antimicrobial susceptibility testing (AST) were carried out using MicroScan Walkaway (Beckman Coulter) or Vitek 2 (bioMérieux). Isolates were sent to the Multidrug Resistant Organism Repository and Surveillance Network (MRSN) for further phenotypic characterization and genome sequencing. Confirmatory AST was performed in the MRSN College of American Pathologists (CAP)-accredited clinical laboratory using the Vitek 2 (card GN AST 71; bioMérieux, NC, USA). In addition, minimum inhibitory concentrations (MICs) of fosfomycin were determined in triplicate using broth microdilution based on Clinical and Laboratory Standards Institute (CLSI) guidelines.

### Whole-genome sequencing and *de novo* assemblies

Isolates were sequenced on a MiSeq benchtop sequencer (Illumina, Inc., San Diego, CA). DNA was extracted using the DNeasy UltraClean microbial kit (Qiagen, Germantown, MD, USA), and libraries were constructed using the Kapa HyperPlus library preparation kit (Roche Diagnostics, Indianapolis, IN, USA) as previously detailed [24]. Samples were sequenced using MiSeq reagent kit v3 (600 cycles; 2×300 bp) (Illumina). Five isolates were selected for single-molecule real-time (SMRT) sequencing using a PacBio RS II instrument (Pacific Biosciences, CA, USA). MiSeq read data were processed with bbduk to trim Illumina adapters and filter reads by quality prior to assembly. Reads falling below an average of 15 Phred score within a 5 bp sliding window and reads shorter than 100 bp were excluded. Filtered reads were assembled *de novo* using Newbler 2.9 and minimum thresholds for contig size and coverage were set at 200 bp and 49.5×, respectively. PacBio read data were assembled *de novo* using a hierarchical genome assembly process (HGAPv3.0). Overlapping contig ends were removed to circularize individual PacBio contigs. Short-read data were mapped to circularized contigs to correct errors.

### Genome annotation, phylogeny and antimicrobial resistance (AMR) gene detection

The genome of index isolate MRSN 546052 was annotated using Prokka 1.14.6 and variant calling was performed by MrSNP-plus 1.0.3 using the index isolate as a reference. Single-nucleotide polymorphism (SNP)-based trees were generated with RaxML-ng using snippy core genome alignment of all 14 isolates, as detailed previously [18]. AMRFinderPlus v3.9.8 (https://www.ncbi.nlm.nih.gov/pathogens/antimicrobial-resistance/AMRFinder/) and ARIBA v2.14.4 [25] were used to identify resistance alleles in all isolates (Table S1, available in the online version of this article).

### Plasmid typing, predicting toxin–antitoxin systems, mapping and construction

Each individual extrachromosomal contig from PacBio long-read sequencing was considered a plasmid and replicon sequences were identified using PlasmidFinder 2.1 [26]. Toxin–antitoxin systems were predicted using the web-based tool TAfinder [27]. Full plasmid sequences were extracted and analysed with a combination of software and online tools. Insertion sequences (ISs) were detected using the ISfinder tool (https://isfinder.biotoul.fr/) [28]. Rearrangements within hybrid plasmids were visualized in Geneious Prime 2020.2.4 and proposed intermediate molecules were constructed with SnapGene Viewer 5.3.1.

## Results

### Patient history and phylogenetic analysis reveal long-term colonization by multidrug-resistant (MDR) *K. pneumoniae*

A 69-year-old patient was hospitalized in India in January 2013 for management of subarachnoid haemorrhage. A month later, the patient was transferred to an acute care hospital in Pittsburgh, PA, USA, and subsequently discharged to a long-term care facility. In March 2013, the patient was readmitted to the same US hospital presenting with a fever and a suspected urinary tract infection (UTI). A urine culture grew the index CRKP (MRSN 546052) and was positive by PCR for *bla*_NDM*-*1,_ *bla*_OXA-232_ and *armA*. The patient was administered oral fosfomycin for treatment of the UTI. No additional NDM-targeting antibiotic was administered through 2015, though positive cultures from surveillance swabs revealed the patient was still colonized with CRKP throughout this period (Table 1). In that time frame, the patient had extensive exposure to antibiotics (most common regimens included piperacillin–tazobactam, ceftolozane–tazobactam, cefepime, ceftriaxone and meropenem) targeting other multidrug-resistant organisms (MDROs). In 2016, the patient had two episodes of line-associated bacteraemia with MDR *K. pneumoniae *, which was successfully treated with line removal and administration of ceftazidime–avibactam. A year later, the patient presented with a recurring *K. pneumoniae* UTI and was treated with oral fosfomycin. From these admissions (2013–2017), a total of 14 isolates from various sites were collected and sent to the MRSN for genome sequencing and analysis (Table 1).

Phylogenetic analysis on the core genome revealed that all 14 isolates were highly genetically related (average distance to nearest neighbour was 3.8 SNPs) (Fig. 1). Relatedness was generally correlated with the date of isolation; the more recent isolates (i.e. post-2016) were more related to each other (average of 1.4 SNPs to nearest neighbour) than they were to the 2013 index isolate (average of 35 SNPs). In June 2016, a cluster of five nearly genetically identical isolates were collected from one respiratory and four blood samples within days of each other. The only other blood isolate (MRSN 546131) was collected in a preceding episode of bacteraemia (February 2016) and was less genetically related to the other blood isolates (average of 18 SNPs) than to the three isolates (average of 7 SNPs) collected from urine cultures throughout 2017 (Fig. 1).

### Variable carriage of AMR genes in serial isolates correlates with phenotypic resistance

The index isolate MRSN 546052 was phenotypically characterized as XDR with resistance to carbapenems, cephalosporins, β-lactam/β-lactamase inhibitor combinations, and all aminoglycosides [14]. Nine of the serial isolates had an identical susceptibility profile, including all blood isolates collected in 2016 (Fig. 1). In contrast, four isolates (blood isolate MRSN 546131 and phylogenetic neighbours MRSN 546195, MRSN 546201 and MRSN 546210) were susceptible to carbapenems and amikacin but retained resistance to cephalosporins and other aminoglycosides. Interestingly, and unlike all other serial isolates, the three most recently collected urine isolates were non-susceptible to tigecycline (Fig. 1).

With the exception of tigecycline, the acquired AMR genes carried by the isolates correlated well with antibiotic susceptibility (Fig. 1). Index isolate MRSN 546052 carried the carbapenemase genes *bla*_NDM-1_ and *bla*_OXA-232_, the 16S methyltransferase gene *armA* and the ESBL gene *bla*_CTX-M-15_. In contrast to *bla*_CTX-M-15_, which was conserved in all isolates, only two carried both carbapenemase genes and, after June 2013, all successive isolates lacked *bla*_OXA-232_. Blood isolate MRSN 546131, which was the first to show susceptibility to carbapenems and amikacin, lacked *bla*_NDM-1_ and *armA,* and both genes were also missing in the three urine isolates sharing the same susceptibility profile and the highest genetic relatedness (Fig. 1). In addition to *bla*_NDM-1_ and *armA,* these four isolates simultaneously lost other resistance markers (Table S1), suggesting that a large region of DNA, most likely of plasmid origin, was missing.

### In-host rearrangement and streamlining of plasmids carrying resistance genes

To investigate plasmid content, five distinct isolates were chosen for PacBio long-read sequencing. In agreement with its initial characterization [13], the index isolate MRSN 546052 carried four plasmids, hereby labelled pMR283.4-NDM (IncFIB–IncHI1B hybrid replicon carrying resistance genes *bla*_NDM-1_ and *armA*), pMR103.7 (IncFIB-like), pMR70.8-CTXM (IncFIA-like plasmid carrying *bla*_CTX-M-15_) and pMR6.1-OXA232 (ColE-like replicon carrying *bla*_OXA-232_), (Figs 1 and S1). However, in this genome assembly of MRSN 546052 a previously undetected small cryptic plasmid (no known replicon) was circularized and named p2.1. A total of five toxin–antitoxin (TA) systems were predicted from these five plasmids: COG5654/5642, GNAT/RHH, RelE/PHD (all three originating in pMR283.4-NDM), HigB/HigA (carried by pMR103.7) and PIN/AbrB (pMR70.8-CTXM) (Fig. S1). No TA systems were predicted in either pMR6.1-OXA232 or p2.1.

Overall, none of the four other isolates analysed shared genetically identical molecules to the plasmids (hereby referred as canonical) in MRSN 546052, with the exception of p2.1, which was conserved in all. In the case of pMR6.1-OXA232, complete plasmid loss was observed in all four isolates analysed and explained the lack of *bla*_OXA-232_ in the majority of isolates from this patient (Fig. 1). In the cases of pMR283.4-NDM, pMR103.7 and pMR70.8-CTXM, hybrid molecules composed of various segments of these three canonical plasmids were detected in isolates MRSN 546113,-122, -140 and -195. Despite being phylogenetic neighbours, sharing the same AST profile, and having been collected at <4-month intervals, MRSN 546113 and -122 were distinct in their plasmid content; while both isolates carried an identical pMR70.8-CTXM (at the gene content level), MRSN 546113 also carried pMR103.7 and a modified pMR283.4-NDM (formed by the additional insertion of a 1.3 kb IS*4321*) hereby named pMR284.7-NDM (Figs 1 and 2a). In contrast, MRSN 546122 lacked a circular pMR103.7 plasmid, but retained its entire genetic content merged with the canonical pMR283.4-NDM, forming a IncFIB–IncHI1B hybrid plasmid pMR390.4-NDM (Figs 1 and 2b). Similar inter- and intra-plasmid rearrangements of canonical pMR70.8-CTXM (*bla*_CTX-M-15_) and pMR283.4-NDM (*bla*_NDM-1_ and *armA*) were observed in isolate MRSN 546140, which carried the hybrid plasmids pMR235.4-NDM (IncFIB–IncHI1B*, bla*_NDM-1_ and *armA*) and pMR122.3-CTXM (IncFIA, *bla*_CTX-M-15_) (Figs 1 and 2c). Finally, MRSN 546195 carried the least plasmid content, both in number of plasmids and total kilobases, as fragments (each carrying their distinct TA systems) of all pMR283.4-NDM, pMR103.7 and pMR70.8-CTXM canonical molecules converged into a single IncFIA–IncFIB mosaic plasmid named pMR206.6-CTXM (Figs 1 and 3). In the process, *bla*_CTX-M-15_ was retained but *bla*_NDM-1_ and *armA* were lost.

### IS*26* drives plasmid rearrangements

Mechanistically, the inter- and intra-plasmids rearrangements that generated the various hybrid plasmids in isolates MRSN 546113,-122, -140 and -195 were likely mediated primarily by IS*26* transposition events. In all cases, the proposed routes for the genesis of each hybrid plasmids involved pMR284.7-NDM (first described in MRSN 546113, Fig. 2a) as an intermediate. In highly genetically related isolate MRSN 546122, plasmid pMR390.4-NDM likely resulted from an IS*26*-mediated intermolecular transposition of pMR284.7-NDM into pMR103.7 (step 2, Fig. 2b), as indicated by IS*26* and associated 8 bp target site duplications (TSDs) observed in pMR390.4-NDM.

In MRSN 546140, hybrid plasmids pMR235.4-NDM and pMR122.3-CTXM were likely created by homologous recombination (step 4, Fig. 2c) of sequences flanked by *merA* and IS*26* shared by both pMR284.7-NDM and pMR70.8-CTXM (first described in MRSN 546113). Further, for p235.4, a trans-oriented intramolecular attack by IS*26* occurred (step 3, Fig. 2c), either subsequent or antecedent to the recombination event, resulting in inversion of the region containing *bla*_NDM-1_ and duplication of IS*26*.

Finally, in MRSN 546195 (susceptible to carbapenems and amikacin) the proposed genesis of the IncFIA–IncFIB hybrid plasmid pMR206.6-CTXM first involved a trans-oriented intramolecular transposition event in pMR284.7-NDM, where a ~63.5 kb segment was inverted by an IS*26*-mediated attack (step 1, Fig. 3). A duplication of the attacking IS*26* and resulting TSD [29] were at the origin of a newly formed composite transposon, highlighted in the proposed intermediate molecule I1 (Fig. 3). At step 2, this transposon appears to have inserted into the canonical IncFIB pMR103.7 (with the remaining backbone of IncFIB–IncHI1B pMR284.7-NDM carrying *bla*_NDM-1_, *armA* and TA system RelE/PHD being lost), where a subsequent cis-oriented intramolecular attack by IS*26* resulted in the loss of a ~36 kb region encoding a type IV secretion system and the associated TSD (step 3 and proposed intermediate molecule I2, Fig. 3). At step 4, PMR70.8_CTXM (IncFIA) is proposed to have inserted into the intermediate molecule I2 by homologous recombination within the *ltrA* gene and yielded the proposed intermediate molecule I3. Finally, an inversion is likely to have occurred between the two homologous Tn*3 tnpA* sites (step 5) to yield hybrid IncFIA–IncFIB pMR206.6-CTXM, which only carried *bla_CTX-M-15_*.

### Within-patient evolution and emergence of fosfomycin and tigecycline resistance

**Fig. 4.**
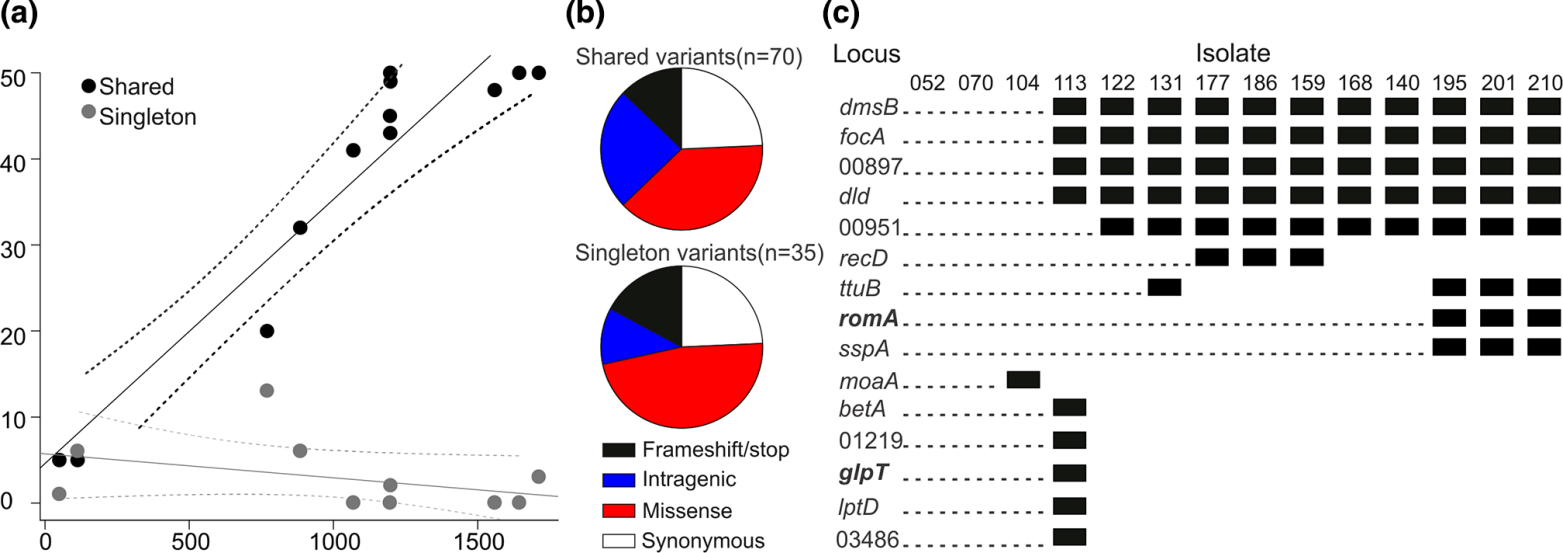
Within-patient evolution and emergence of variants associated with fosfomycin and tigecycline resistance. (**a**) Count of shared (in ≥2 isolates and using MRSN 546052 as a reference) or singleton SNPs in serial isolates over time (*x*-axis, in days since collection of index isolate). (**b**) Distribution of predicted impact based on annotation and stratified by shared and singleton variants. (**c**) Distribution of shared and singleton high-impact variants (frameshift, large indels, stop codon) in all 14 serial isolates (ordered by date of isolation). Presence of a variant is indicated by a black box. Corresponding gene names or locus tags are indicated and genes with known association with AMR are indicated in bold.

Whole-genome variant detection and annotations were performed to provide a more comprehensive overview of the evolution of this clone of *K. pneumoniae *. Variants were classified as singleton (observed once) or shared (observed more than once) across the 14 isolates. For each isolate, the total number of singleton or shared SNPs (i.e. excluding indels) was plotted as a function of days, since the index culture and linear regression showed an accumulation of shared variants at ~11 SNPs per year (Fig. 4a), similar to rates previously described for *K. pneumoniae* [21, 30]. Seventy shared variants were identified in addition to 35 singleton variants (Fig. 4b and Table S2). The distribution of the predicted variant impact was similar between shared and singletons with the majority (~40 %) being missense mutations and 12–15 % being predicted loss-of-function (LOF) mutations. Specifically, a total of 15 genes carried either a frameshift or a stop codon mutation in ≥1 isolates (Fig. 4c). Predicted LOF due to mutations in the genes coding for a subunit of the dimethyl sulfoxide reductase (*dmsB*), a formate–nitrine transporter (*focA*), a putative lipoprotein (00897), a d-lactate dehydrogenase (*dld*) and a putative pseudocatalase (00951) were acquired early (first detected in June 2015) and shared by all isolates thereafter. While the impact of these variants remains unclear, a one-nucleotide substitution in *romA* (part of the *romA–ramA* locus), resulting in a premature stop codon, was observed in the three most recent isolates and was likely responsible for their acquired phenotypic resistance to tigecycline via upregulation of an efflux-mediated pathway, as described previously [31] (Figs 1 and 4c). Similarly, five predicted LOF mutations were uniquely found in isolate MRSN 546113 (collected from a rectal swab in April 2015), including a frameshift in *glpT* that encodes the main transporter responsible for the uptake of fosfomycin (Fig. 4c) [32]. While this mutation possibly emerged as a result of the oral fosfomycin regimen prescribed on at least four different occasions between 2013 and 2017 (Table 1), no noticeable phenotypic impact was observed *in vitro*, as all 14 isolates were highly resistant to this antibiotic (MIC >256 µg ml^−1^) due to the presence of a conserved chromosomal *fosA* allele.

## Discussion

In this study, longitudinal sampling of a chronically infected patient showed that a ST14 CRKP clone, initially harbouring *bla*_NDM-1_ and *bla*_OXA-232_ on separate plasmids, rapidly lost both carbapenemases through complete plasmid loss and IS*26*-mediated plasmid reshuffling, eventually restoring carbapenem susceptibility. This echoes previous observations that plasmids carrying AMR genes generally impose a fitness cost on their bacterial hosts in the absence of selective pressure [21, 33, 34]. Specifically, the plasticity of *K. pneumoniae* plasmids has been extensively described using global collections or serially passaged isolates *in vitro* [35, 36]. Recently, additional reports on the evolution of plasmids within the host are emerging [20–22, 37]. For example, in 2017, an analysis of 15 *K *. *pneumoniae* isolates from six patients revealed the loss of *bla*_KPC_ in one patient, and an overall significant reduction in plasmids due to recombination events in isolates from multiple patients [20]. Other studies, generally focused on serial *K. pneumoniae* isolates from patients with chronic infections and prolonged length of stay, have also reported decreased copy number or complete loss of plasmids in the absence of antibiotic pressure [21, 34].

Acknowledging this large body of evidence for the biological cost of carrying plasmids, the ‘plasmid paradox’ was theorized to represent the evolutionary dilemma posed by the persistence, over the long term, of conjugative plasmids instead of the fixation of the select beneficial genes into the bacterial chromosome [38, 39]. In such a context, the repeated identification of *K. pneumoniae* isolates carrying plasmid-borne *bla*_NDM-1_ and *bla*_OXA-232_ carbapenemases [5–7, 14] appears even more paradoxical. Interestingly, a recent study showed that the simultaneous presence of a large *bla*_NDM-1_ (IncFIB/IncHI1B) and a smaller *bla*_OXA-232_ (ColE-like) plasmid, sharing a high level of genetic relatedness with pMR283.4-NDM and pMR6.1-OXA232 analysed here, increased the fitness and virulence of a transconjugant *Escherichia coli* host *in vitro*, while a single plasmid did not [40]. This is reminiscent of observations from San Millan *et al.* who showed that co-infection of a large and a small plasmid could invoke positive epistasis, minimizing the cost associated with carrying multiple plasmids [41] and could contribute to their relatively high prevalence in nature.

An important element in the persistence and stability of plasmids is toxin–antitoxin systems [42, 43] and our observation of a rapid, complete loss of plasmid pMR6.1-OXA232 (the only molecule lacking a TA system) in these ST14 isolates further illustrates that. Interestingly, TA systems harboured by all the other AMR-carrying plasmids did not prevent the loss of *bla*_NDM-1_ and *armA* in isolate MRSN 546195. Instead, all TA systems (with the exception of RelE/PHD, which is likely explained by cross-reactivity with the antitoxin from HigB/HigA [44]) were conserved via the convergence of fragments of their respective canonical plasmids into the mosaic pMR206.6-CTXM molecule.

The detailed molecular analysis of circularized plasmid sequences from serial isolates collected in this study revealed that IS*26* is a major contributor to the genetic reshuffling of carbapenemase-carrying plasmids. The IS*26* family is common in carbapenem-resistant *Enterobacterales* [45] and has been implicated in plasmid reorganization [29] through well-characterized replicative and/or conservative transposition mechanisms [29, 46, 47]. ISs are well known to introduce adaptive traits into plasmids and a recent study even characterized IS*26*-flanked pseudo-composite transposons as the likely primary contributor to the genetic reshuffling of *bla*_NDM_ [16, 48]. However, the work by Porse *et al.* also showed that IS*26* is a driving force of plasmid persistence in novel hosts [35]. Specifically, they showed that IS*26*-mediated deletions of costly regions, such as type 4 secretion systems (T4SSs), from a plasmid backbone could improve plasmid stability and effectively expand its host range [49]. Interestingly, in our study, genes coding for a T4SS were also lost in the intermolecular and intramolecular shuffling, leading to the emergence of plasmid pMR206.6-CTXM. Consistent with our observations, such restructuring events are thought to come at the expense of conditionally useful components (e.g. the loss of *armA* and *bla*_NDM-1_) that might ultimately limit plasmid dissemination.

A few considerations for this study should be noted. First, our observations are derived from selective culture (i.e*.* single colonies) of serial rectal surveillance swabs and other clinical specimens, which risks underestimating diversity [50], including the possible co-carriage of carbapenem-resistant and carbapenem-susceptible ST14 strains within or between the different body sites. In fact, this could explain an apparent resurgence of CRKP cultures in January 2018 in this patient (unfortunately isolates were not available for sequencing), after all urine isolates collected in 2017 lacked *bla*_NDM-1_ and *bla*_OXA-232_. Second, although exposure to tigecycline was not documented, the patient’s medical history while outside of the hospital (e.g*.* while at a LTC facility) is unknown. It cannot be excluded that additional antibiotic regimens were prescribed and favoured the emergence of tigecycline resistance via the inactivation of *romA*, a mutation known to arise in response to treatment [51, 52]. Third, while congruent with observations reported in other studies [8, 29, 34, 35, 53], the proposed routes for plasmid streamlining observed in these isolates from a single patient likely only represent a partial picture of the many ways IS elements, and IS*26* in particular, drive plasmid evolution by maintaining genetic plasticity.

As such, further investigations of the mechanisms underlying plasmid persistence and the role of transposable elements herein are needed to understand and prevent our current epidemic of multidrug resistance. As demonstrated here, the elucidation of the complex repetitive plasmid patterns is now achievable using long-read sequencing. The advancement of these technologies opens up many avenues from the assessment of plasmid maintenance, spread and diversity, to the integration of such efforts into global and local surveillance programmes.

## Supplementary Data

Supplementary material 1
