# Supplementary material for: IS26-mediated plasmid reshuffling results in convergence of toxin–antitoxin systems but loss of resistance genes in XDR Klebsiella pneumoniae from a chronic infection
Source: Microb Genom. 2022 Sep 28;8(9):mgen000892. doi: 10.1099/mgen.0.000892 (PMC9676029; doi:10.1099/mgen.0.000892)
Supplement: Supplementary material 1 [file mgen-8-892-s001.pdf]

SUPPLEMENTARY MATERIAL

IS*26*-mediated plasmid reshuffling results in convergence of toxin-antitoxin systems but loss of resistance genes in XDR *Klebsiella pneumoniae* from a chronic infection

Ting L. Luo[1*], Brendan W. Corey[1*], Erik Snesrud[1*], Alina Iovleva[2], Christi L. McElheny[2], Lan Preston[1], Yoon Kwak[1], Jason W. Bennett[1], Yohei Doi[2], Patrick T. McGann[1] and Francois Lebreton[1#]

[1] Multidrug-Resistant Organism Repository and Surveillance Network (MRSN), Walter Reed Army Institute of Research, Silver Spring, Maryland, USA

[2] Division of Infectious Diseases, University of Pittsburgh School of Medicine, Pittsburgh, Pennsylvania, USA.

*Authors contributed equally.

#Address correspondence to francois.lebreton.ctr@mail.mil

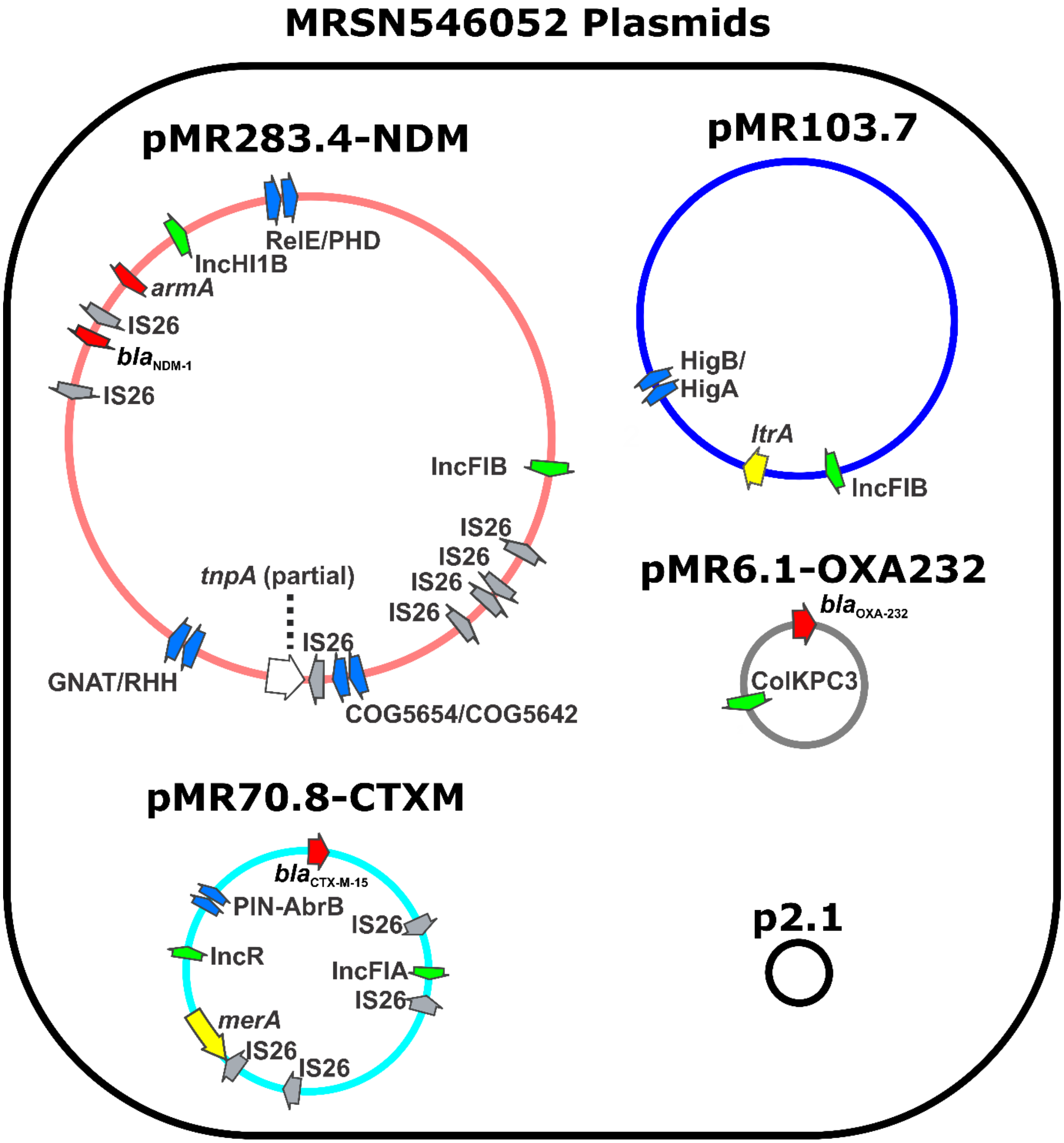

Figure S1: Plasmid profile of index isolate MRSN546052. Five plasmids were detected in the index isolate using PacBio long read sequences. Segments of canonical plasmids are rendered according to their assigned color in figure 1. Antimicrobial resistance genes (red), insertion sequences (gray), plasmid replicon ori sites (green), and putative transposases (white) are indicated as well as genes at the boundary of recombination or insertion events (yellow).

Table S1: Presence and absence of antibiotic resistance genes in 14 *K. pneumoniae*.

	546052	546104	546070	546113	546122	546140	546159	546168	546177	546186	546131	546195	546201	546210
aac(3)-IId	+	+	+	+	+	+	+	+	+	+	+	+	+	+
aac(6')-Ib3	+	+	+	+	+	+	+	+	+	+	+	+	+	+
aac(6')-Ib-cr	+	+	+	+	+	+	+	+	+	+	+	+	+	+
aadA2	+	+	+	+	+	+	+	+	+	+	-	-	-	-
aph(3'')-Ib	+	+	+	+	+	+	+	+	+	+	+	+	+	+
aph(3')-VI	+	+	+	+	+	+	+	+	+	+	-	-	-	-
aph(6)-Id	+	+	+	+	+	+	+	+	+	+	+	+	+	+
armA	+	+	+	+	+	+	+	+	+	+	-	-	-	-
arr-2	+	+	+	+	+	+	+	+	+	+	+	+	+	+
$bla_{CTX-M-15}$	+	+	+	+	+	+	+	+	+	+	+	+	+	+
bla_{NDM-1}	+	+	+	+	+	+	+	+	+	+	-	-	-	-
bla_{OXA-1}	+	+	+	+	+	+	+	+	+	+	+	+	+	+
blaOXA-232	+	+	-	-	-	-	-	-	-	-	-	-	-	-
bla_{OXA-9}	+	+	+	+	+	+	+	+	+	+	+	+	+	+
bla_{TEM-1A}	+	+	+	+	+	+	+	+	+	+	+	+	+	+
catB3	+	+	+	+	+	+	+	+	+	+	+	+	+	+
cmlA1	+	+	+	+	+	+	+	+	+	+	+	+	+	+
dfrA1	+	+	+	+	+	+	+	+	+	+	+	+	+	+
dfrA12	+	+	+	+	+	+	+	+	+	+	-	-	-	-
dfrA14	+	+	+	+	+	+	+	+	+	+	-	-	-	-
ere(A)	+	+	+	+	+	+	+	+	+	+	+	+	+	+
fosA	+	+	+	+	+	+	+	+	+	+	+	+	+	+
mph(E)	+	+	+	+	+	+	+	+	+	+	-	-	-	-
msr(E)	+	+	+	+	+	+	+	+	+	+	-	-	-	-
oqxA	+	+	+	+	+	+	+	+	+	+	+	+	+	+
oqxB	+	+	+	+	+	+	+	+	+	+	+	+	+	+
qnrB1	+	+	+	+	+	+	+	+	+	+	-	-	-	-
sul1	+	+	+	+	+	+	+	+	+	+	+	+	+	+
sul2	+	+	+	+	+	+	+	+	+	+	+	+	+	+

Table S2: Presence and absence of all genetic variations observed in 14 *K. pneumoniae*.

	546052	546070	546104	546113	546122	546131	546177	546186	546159	546168	546140	546195	546201	546210
ybhR		+	+	+	+	+	+	+	+	+	+	+	+	+
ydeP		+	+	+	+	+	+	+	+	+	+	+	+	+
narK				+	+	+	+	+	+	+	+	+	+	+
narK				+	+	+	+	+	+	+	+	+	+	+
panF				+	+	+	+	+	+	+	+	+	+	+
deoB				+	+	+	+	+	+	+	+	+	+	+
tehA				+	+	+	+	+	+	+	+	+	+	+
01558				+	+	+	+	+	+	+	+	+	+	+
dnaX				+	+	+	+	+	+	+	+	+	+	+
cmoB				+	+	+	+	+	+	+	+	+	+	+
01692				+	+	+	+	+	+	+	+	+	+	+
pflD				+	+	+	+	+	+	+	+	+	+	+
dld				+	+	+	+	+	+	+	+	+	+	+
00897				+	+	+	+	+	+	+	+	+	+	+
focA				+	+	+	+	+	+	+	+	+	+	+
dmsB				+	+	+	+	+	+	+	+	+	+	+
mdtM					+	+	+	+	+	+	+	+	+	+
msrP					+	+	+	+	+	+	+	+	+	+
maoA					+	+	+	+	+	+	+	+	+	+
arcA					+	+	+	+	+	+	+	+	+	+
ansP					+	+	+	+	+	+	+	+	+	+
00951					+	+	+	+	+	+	+	+	+	+
noc					+	+	+	+	+		+	+	+	+
05217					+		+	+	+	+	+			
lptD							+	+	+	+	+			
02224							+	+	+	+	+			
metR							+	+	+	+	+			
yajO							+	+	+	+	+			
moxC							+	+	+	+	+			
01222							+	+	+	+	+			
sdaC							+	+	+	+	+			
aaeB							+	+	+	+	+			
oppF							+	+	+	+	+			
der							+	+	+	+	+			
bgiY							+	+	+	+	+			
namA						+						+	+	+
aceE						+						+	+	+
gsk						+						+	+	+
rpsD						+						+	+	+
dadA						+						+	+	+
rsxE						+						+	+	+
ttuB						+						+	+	+
tmcA												+	+	+
phoC												+	+	+
cobS												+	+	+
sspA												+	+	+

	**546052**	**546070**	**546104**	**546113**	**546122**	**546131**	**546177**	**546186**	**546159**	**546168**	**546140**	**546195**	**546201**	**546210**
00338												+	+	+
recD							+	+	+					
05141													+	+
05134													+	+
04047									+		+			
01697									+		+			
01630									+		+			
05257		+												
moaA			+											
setA			+											
uhpT			+											
dmlR			+											
05134			+											
betA				+										
01219				+										
glpT				+										
lptD				+										
03486				+										
nuoK				+										
edd				+										
malK				+										
ppnN				+										
menH				+										
acnA				+										
dgoT				+										
selB					+									
proA					+									
lldP					+									
mipA					+									
trkA					+									
ccmB					+									
xanP									+					
ndmA									+					
ycfH										+				
pheP											+			
03762											+			
outO														+
bgaA														+
